# A Preference for a Sexual Signal Keeps Females Safe

**DOI:** 10.1371/journal.pone.0000422

**Published:** 2007-05-09

**Authors:** Tae Won Kim, John H. Christy, Jae C. Choe

**Affiliations:** 1 School of Biological Sciences, Seoul National University, Seoul, Korea; 2 Naos Laboratory, Smithsonian Tropical Research Institute, Balboa, Ancón, Panamá; 3 Graduate Program in EcoScience, Ewha Womans University, Seoul, Korea; University of Exeter, Cornwall Campus, United Kingdom

## Abstract

Predation is generally thought to constrain sexual selection by female choice and limit the evolution of conspicuous sexual signals. Under high predation risk, females usually become less choosy, because they reduce their exposure to their predators by reducing the extent of their mate searching. However, predation need not weaken sexual selection if, under high predation risk, females exhibit stronger preferences for males that use conspicuous signals that help females avoid their predators. We tested this prediction in the fiddler crab *Uca terpsichores* by increasing females' perceived predation risk from crab-eating birds and measuring the attractiveness of a courtship signal that females use to find mates. The sexual signal is an arching mound of sand that males build at the openings of their burrows to which they attract females for mating. We found that the greater the risk, the more attractive were males with those structures. The benefits of mate preferences for sexual signals are usually thought to be linked to males' reproductive contributions to females or their young. Our study provides the first evidence that a female preference for a sexual signal can yield direct survival benefits by keeping females safe as they search for mates.

## Introduction

The strength of sexual selection is generally thought to decrease as the risk of predation during courtship increases and constrains both male signalling and female choice [1], [2], [3]. Indeed, many studies have shown that the degree of female choosiness and extent of mate-searching behaviour decrease with increasing predation risk [4], [5], [6], [7], [8]. This may be because the cost of predation exceeds the benefits of highly selective mate choice.

However, females that leave the security of familiar territory to find a mate [9] may continue to be highly mate selective if they prefer to visit courting males that use signals that help females avoid their predators. Such a preference would favour the evolution of signals permitting safe mate searching even under elevated predation risk. Here we test this predicted [10] but little studied function of a female preference by increasing females' perceived predation risk and measuring the attractiveness of male fiddler crabs that use signals that females approach to escape predators and to find mates.

**Figure 1:**
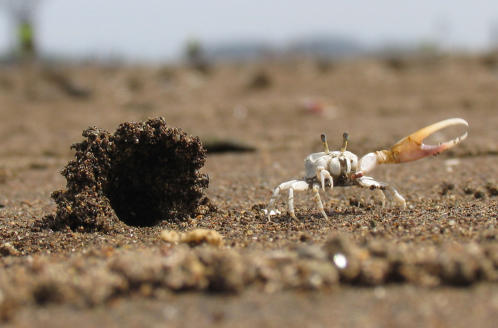
A male fiddler crab *Uca terpsichores* with a sand hood as a sexual signal. The male waves its enlarged claw to attract females to its burrow which has a sand hood at the entrance. Males are about 1 cm wide. Hoods project above the visual horizon of crabs. This makes them conspicuous landmarks that help crabs find burrows.

Courting males of 18 of the 97 species in the fiddler crab genus *Uca* sometimes build structures of mud or sand at the openings of their intertidal burrows wherein crabs mate [11]. Female crabs of these structure-building species leave their own burrows and visit from a few up to 100 courting (claw-waving, Fig. 1) males [12] before they stay with one in his burrow, mate and breed. Females of at least two species of fiddler crabs prefer to visit males that build structures [11], [13], [14]. Mate-searching females are at risk of predation from shorebirds as they move between males' burrows and they stop searching when birds are exceptionally abundant [15]. Females without access to burrows approach and hide against male-built structures and other objects on the sand both spontaneously and when chased experimentally by a replica of a crab-eating bird [13], [14], [16]. These observations suggest that the preference for male-built structures may help females escape predation while they seek mates.

We tested this function for the female preference for visiting hood-building males of the fiddler crab *Uca terpsichores* by determining whether the strength of the preference increased with perceived predation risk. We attracted Great-tailed Grackles with bait to one of two adjacent, visually isolated areas with crabs, alternating daily the area baited for birds. We measured attractiveness as the proportion of courtships in which females visited males with and without hoods that directed claw waving to them. Perceived predation risk in an area was the number of times each day that birds caused crabs, including the mate-searching females, to hide in burrows.

## Results

**Figure 2:**
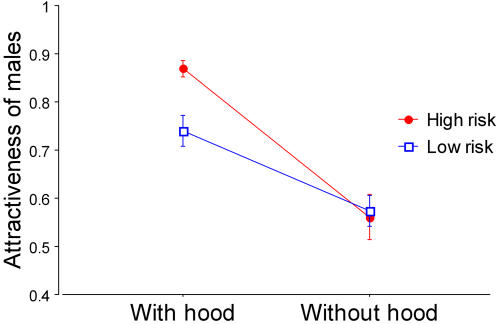
The effect of interaction between hood presence and predation risk treatment on male attractiveness. The attractiveness (female visits/visits+passes, mean±SE) of males with hoods was higher than that of males without hoods regardless of treatment. Under high predation risk (red circle) males with hoods were more attractive than they were under low predation risk (blue square). The attractiveness of males without hoods was not influenced by risk treatment.

**Figure 3:**
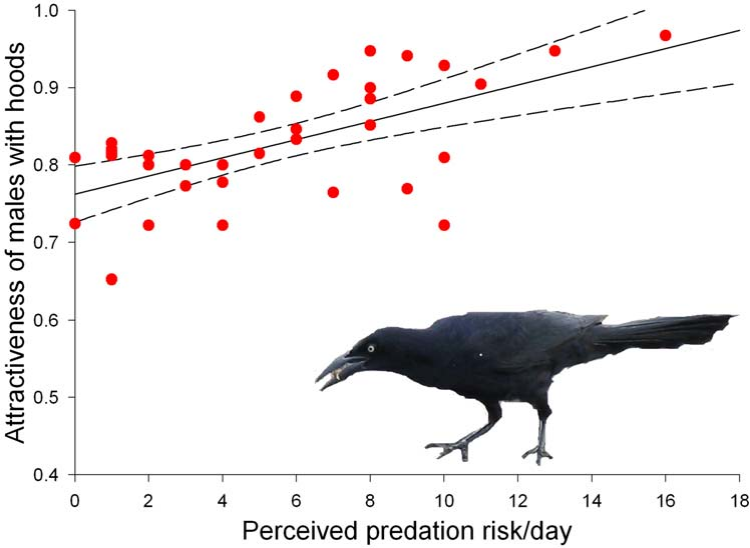
The attractiveness of males with hoods in relation to level of perceived predation risk. The attractiveness of males with hoods increased significantly with increasing level of perceived predation risk (number of times grackles caused female crabs to retreat to their burrows/day; *n* = 34, *r* ^2^ = 0.379, *t* = 4.418, *p*<0.0001, excluding two outliers; 95% confidence limits shown.

Male fiddler crabs with hoods were always more attractive than those without hoods regardless of predation risk treatment (high or low). There was a significant interaction between the attractiveness of males with and without hoods and predation risk treatment (Table 1 and Fig. 2). Males with hoods were more attractive in the areas with high predation risk than in the areas with low predation risk (*F*_1,34_ = 12.515, *p* = 0.001). The attractiveness of males without hoods did not depend significantly on risk treatment (*F*_1,34_ = 0.056, *p* = 0.814). With one exception, the total number of visits per courtship to males with hoods was also significantly higher in the areas with high predation risk than in the areas with low predation risk (Table 2). Most notably, the attractiveness of males with hoods increased linearly and significantly with the level of perceived predation risk (Fig. 3). These results support a risk-reducing function for the preference for visiting males with hoods.

**Table 1:** Nested ANOVA table of the effects of predation risk treatment, presence of a hood, paired area combination and risk treatment order on male attractiveness

Factor or interaction	df	Sum of Square	Mean Square	*F*	*p*
Predation risk	1	0.060	0.060	3.07	0.085
Hood	1	1.017	1.017	51.84	<0.001
Predation risk×Hood	1	0.092	0.092	4.68	0.034
Area combination	2	0.040	0.020	0.47	0.664
Risk treatment order (Area combination)	3	0.128	0.128	2.18	0.099
Error	63	1.236	0.020		
Total	71	2.574			

**Table 2:** The attractiveness of males with or without hoods under high and low levels of perceived predation risk in three different areas

Area	Male category	Predation risk	Visits/courtships	*G*	*p*
A	With hood	High	140/152	11.331	<0.001
		Low	163/206		
	Without hood	High	36/72	1.696	0.192
		Low	60/100		
B	With hood	High	76/92	8.280	0.004
		Low	63/98		
	Without hood	High	70/129	0.123	0.725
		Low	108/192		
C	With hood	High	107/121	2.469	0.116
		Low	139/170		
	Without hood	High	38/74	0.325	0.568
		Low	59/106		

## Discussion

Females of many species are attracted to mate with males that use certain courtship signals. The benefits of such preferences usually are thought to derive from the signalling male's ability to contribute directly or indirectly to female fitness. Direct benefits include increasing the female's fertility or fecundity [17], perhaps by providing access to superior resources [18], or providing care to her offspring [17]. Indirect benefits include genetic contributions to the viability or reproductive success of the female's young [9], [19], [20]. Our results do not exclude these possible reproductive benefits but suggest that females may also gain a direct survival (non-reproductive) benefit from a preference for a sexual signal; as predation risk increased so too did the strength of the preference that helps females avoid predators.

We believe that this is the first demonstration that predation risk increases the attractiveness of a conspicuous male courtship signal because the signal elicits predator avoidance behaviour in females. This case contrasts with those of many other animals in which predation risk decreases the use of and preferences for highly conspicuous courtship signals [4], [5], [6], [7] or reduces the extent or frequency of mate searching [8]. Our discovery of a risk-reducing mate preference in *Uca terpsichores* may be explained by the high risk of predation to which females are exposed every time they leave the safety of one male's burrow and move to the next. Great-tailed Grackles frequently eat fiddler crabs at the study site [15] and we saw them eat *U. terpsichores* during this study. However, we did not see a single case of predation by this or other species of crab-eating birds on mate-searching female *U. terpsichores*. The high survivorship of mate searching females is testament to the efficiency with which females avoid their predators. Indeed, orientation to sand hoods is one of several traits that may keep mate searching females safe. Other risk-reducing traits of fiddler crabs include females' highly cryptic colour pattern, the formation and use of a non-visual path map to the burrow they have just left, and orientation to the visual image of a courting male crab as it disappears into its burrow [21], [22]. Other examples of preferences for sexual signals that keep females safe may be found in other animals in which females that lack chemical or structural defences evaluate and choose mates under conditions of high predation risk.

Warner and Dill [10] proposed that female bluehead wrasse (*Thalassoma bifasciatum*) use conspicuous signalling by brightly coloured males to judge when it is safe to visit the male for spawning, an act that briefly exposes both sexes to an increased risk of predation. According to this “safety assurance” hypothesis, the signalling behaviour of male wrasse conveys information about the safety of the site. In contrast, fiddler crab structures do not convey information about the predation risk of courtship or mating at a particular time or location. Instead, crab structures facilitate female predator avoidance behaviour. As we have shown here, crab structures are especially attractive when predation risk to mate-searching females is relatively high.

Our study provides a striking example of how female assessment of an environmental factor (predation risk) affects the expression of an adaptive (predator avoidance) mate preference and contributes to variation in female mate choice [23]. As the relative frequency of female visits to males with structures increases, so does the relative mating success of structure building males [11], [13], [14]. Through this mechanism, predation risk should strengthen sexual selection for signal-preference pairs that increase male mating rates because they help mate-searching females avoid their predators. Variation among the 97 known species of fiddler crabs in how females seek mates and their predation risk while searching may have contributed to the evolution of the diverse forms of courtship in this highly sexually selected genus [24].

## Materials and Methods

### Study site and species

We observed crabs from December 2005 through February 2006 on an intertidal sand flat on the west bank of the Pacific entrance to the Panama Canal about 1 km upstream of the Bridge of the Americas (8° 56′N 79° 34′W). *U. terpsichores* lives in burrows, one crab per burrow (except for mating pairs) in a mixed-sex population on the flat. Crabs are active on the surface during the day when the tide exposes their habitat. Great-tailed Grackles, *Quiscalus mexicanus,* primarily males, are the most common avian predator of *U. terpsichores* at the study site. Mate-searching female fiddler crabs avoid grackles by running into the burrows of courting males.

### Experimental design

We increased the crabs' perceived predation risk by attracting grackles with a handful of dry dog food scattered on the sand flat. Three, 5 m×8 m areas, each with from 100–400 courting male *U. terpsichores,* were designated areas A, B, and C. The number of hood-building males in each area varied daily from about 50–200. The areas were at least 10 m apart, far enough so that the crabs in a given area did not startle in response to predators that were attracted to one of the other areas.

The experiment was done during three periods of 6 consecutive days corresponding to the days in each biweekly activity cycle that crabs do most of their courtship and mating. We used areas A and B during the first period, areas B and C during the second, and areas A and C during the third. We waited to attract birds each day until after most males had built their hoods so that the birds would not affect male hood building. Each day, 30 min before the time of low tide, grackles were attracted to one of the areas of a pair. The following day, grackles were attracted to the other area of the pair and so on for each pair of areas and 6-day observation period.

### Observation

Each day we observed crabs in each area for two 30-min intervals, starting with one area then switching between them until 2 hours had elapsed. We observed the areas in the same sequence for two days then switched the sequence. Grackles sporadically came to the areas and fed on the dog food and the crabs. In response to the presence of these predators the crabs ran into their burrows. We used the number of times in the 30 min observation period that crabs hid in their burrows in response to predators as a measure of their perceived predation risk.

We observed mate-searching females through binoculars while sitting still in chairs about 5 m from an area. We recorded the responses of a given female to courtship from 2 to 6 males and then shifted our attention to a new female. Lack of strict independence in successive observations of the same individual female does not bias measurement of the attractiveness of males with and without hoods [11]. We judged that courtship occurred when a male clearly directed claw waving toward the female. We scored a visit when the female stopped at the male's burrow and a pass when she walked by without touching the burrow. The attractiveness of males with hoods or without hoods was the frequency of visits to males with or without hoods divided by the frequency of courtships from these males.

### Statistical analysis

We used a GLM nested ANOVA design to test for significant effects of predation risk treatment (high and low), presence of hood, paired area combination (AB, AC, BC) and risk treatment order (Low risk - High risk, High risk - Low risk) on male attractiveness. Treatment order was nested within the three unique paired area combinations. Predation risk and hood presence were fixed factors. Area combination and treatment order were random factors. We found no significant effects of paired area combination or treatment order on attractiveness. We therefore used an ANOVA to determine if predation risk influences the attractiveness of males with or without hoods. We also used a *G*-test of goodness-of-fit to compare the proportion of female visits to courting males for six days at each area under low and high predation risk. The expected frequencies of visits were based on the relative frequencies of courtships by males with and without hoods. We also plotted the attractiveness of males with hoods each day against the level of predation risk to see if there was a significant positive relationship between these two variables.
